# Comparative analysis of asexual and sexual stage *Plasmodium falciparum* development in different red blood cell types

**DOI:** 10.1186/s12936-020-03275-9

**Published:** 2020-06-05

**Authors:** Linda E. Amoah, Festus K. Acquah, Prince B. Nyarko, Elizabeth Cudjoe, Dickson Donu, Ruth Ayanful-Torgby, Fredericka Sey, Kim C. Williamson, Gordon A. Awandare

**Affiliations:** 1 Department of Immunology, Noguchi Memorial Institute for Medical Research, University of Ghana, Accra, Ghana; 2 West African Centre for Cell Biology of Infectious Pathogens, University of Ghana, Accra, Ghana; 3 Ghana Institute of Clinical Genetics, Korle-Bu, Accra, Ghana; 4 Microbiology and Immunology Department, Uniformed Services University of the Health Sciences, Bethesda, MD USA

**Keywords:** Haemoglobinopathies, Malaria, Gametocyte, Merozoite

## Abstract

**Background:**

Red blood cell (RBC) polymorphisms are suggested to influence the course of *Plasmodium falciparum* malaria. Whereas some variants have been found to be protective, others have been found to enhance parasite development. This study evaluated the effect of variant haemoglobin (Hb) and ABO blood groups on *P. falciparum* merozoite invasion, multiplication rates as well as gametocyte development.

**Methods:**

Approximately 2.5 mL of venous blood was collected from each participant. Flow cytometry was used to determine the in vitro merozoite invasion rates of NF54 parasites into the blood of 66 non-parasitaemic individuals with variant Hb genotypes (HbSS, HbSC) and blood groups (A, B, O), which were then compared with invasion into HbAA blood. The ex vivo asexual parasite multiplication and gametocyte production rates of parasites from 79 uncomplicated malaria patients with varying Hb genotypes (HbAS, HbAC and HbAA) were also estimated using microscopy.

**Results:**

Merozoite invasion rates were significantly reduced by about 50% in RBCs containing HbSS and HbSC relative to HbAA cells. The presence of blood group O and B reduced the invasion rates of HbSS by about 50% and 60%, respectively, relative to HbSC but the presence of blood group A removed the inhibitory effect of HbSS. The initial parasite densities in uncomplicated malaria patients with Hb genotypes HbAS and HbAC cells were similar but significantly lower than those with genotype HbAA. The ex vivo parasite multiplication rate, gametocytaemia and gametocyte conversion rates followed a similar trend but did not reach statistical significance (p > 0.05).

**Conclusions:**

Parasite invasion rate into erythrocytes is dependent on both erythrocyte blood group antigen and haemoglobin genotype as blood group O and B provided protection via reduced merozoite invasion in RBCs containing HbSS relative to HbSC. Regardless of haemoglobin type, greater than 70% malaria patients had circulating ring stage parasites that differentiated into stage II gametocytes in 4 days.

## Background

The selective force of *Plasmodium* infections in malaria-endemic countries has resulted in human resistance to the disease. The main genetic determinants of this resistance include glucose-6-phosphate dehydrogenase (G6PD) deficiency [1], thalassemias [2], haemoglobinopathies (beta haemoglobin gene variants [3–5]) as well as blood group ‘O’ [6].

The beta haemoglobin variants that offer protection against malaria include HbC, which is an abnormality in the β-globin subunit of the haemoglobin gene causing the glutamic acid residue at position 6 to be replaced with a lysine residue [4, 5], and HbS [3], in which the same glutamic acid is substituted with valine. Individuals who are homozygous for HbCC have been found to be strongly protected against severe malaria, while heterozygous HbAC individuals were found to be only mildly protected [7]. With HbS, the homozygous state is associated with severe complications, which usually results in death [8]. However, the heterozygous state, HbAS is associated with protection from severe as well as uncomplicated malaria [7, 9, 10] and hospitalization due to malaria [11].

Although a number of studies have reported of the protective effects of variant HbC and HbS, only a few have provided mechanisms that justify the associations [12]. While mechanisms such as RBC structural changes and its impact on disease progression and pathology have been proven [13–15], some other mechanisms including the impaired trafficking of PfEMP1 on the infected RBC surface [16] and activation of heme oxygenase by HbS [17] have been reported but not validated [18].

All ages of human red blood cells can be invaded by *Plasmodium falciparum* merozoites [19]. The invasion process has been studied directly using live microscopy [20, 21] and indirectly by determining the number of newly infected RBCs with ring-stage parasites using light or fluorescent microscopy [22, 23] and flow cytometry-base assays [24–27]. Successful invasion of RBCs by a merozoite results in the initiation of the erythrocytic life cycle of the parasite, where both asexual and sexual stage (gametocytes) parasites develop [28–30]. Merozoite invasion rates have been positively correlated with haematocrit [31], while red blood cell (RBC) polymorphisms including surface antigen diversity have been found to reduce the efficiency of merozoite invasion [19, 32].

*Plasmodium falciparum* gametocytes, which develop through five stages within the RBC, the early stages (stage I–II) and the late stages (stage III–V) [33] are derived from the asexual parasite. Only mature stage V gametocytes circulate in peripheral blood, while the immature stages are sequestered primarily in the bone marrow [34]. Stage V gametocyte density has been found to be associated with anaemia [35] and the presence of RBCs containing HbC and HbS [36]. However, a recent work that quantified gametocyte committed ring stage parasites in uncomplicated malaria patients indicated that gametocytaemia was associated positively with parasitaemia and negatively with fever but not haemoglobin levels [37]. Haemoglobin genotype was however not assessed.

Asexual parasite replication usually results in the presentation of signs and symptoms of malaria in the infected hosts (symptomatic infections). However, other infections can remain asymptomatic. Similarly, sexual stage parasite (gametocyte) development within the host’s erythrocyte is not associated with disease pathology. Asymptomatic parasite carriage (both asexual and gametocyte) is a huge burden to malaria control as the host remains afebrile and does not seek treatment. As global efforts move from malaria control towards elimination and global eradication, identifying gametocyte carriage and factors that influence malaria transmission has become a necessity [38]. With haemoglobin variants suggested to enhance malaria transmission [36], malaria control strategies will benefit from knowledge on how these variants affect parasite growth and gametocyte carriage.

To determine the contribution of host RBC polymorphisms on the establishment and transmission of malaria, this study explored *P. falciparum* merozoite invasion and multiplication rates, as well as gametocyte development in RBCs containing variant β-globin (Hb) genotypes and ABO blood groups. Significant differences in merozoite invasion were observed between the variant Hb genotypes as well as among the three major blood groups. Parasite multiplication rates and gametocyte production rates were however not significantly different among the variant Hb genotypes tested.

## Methods

### Ethical statement

The Institutional Review Board of the Noguchi Memorial Institute for Medical Research (NMIMR), University of Ghana provided approval for the study (protocols # 085/12-13 and 024/14-15). Prior to enrollment, all study participants, including men, women and children were educated on the purpose of the study. Afterwards, a written informed consent, assent and parental consent were obtained from the participants and/or their parents (guardians).

### Study site and population

Samples for the study were obtained from two cohorts; one comprised of a group of children with uncomplicated malaria (UM) and the other comprised of afebrile participants with haemoglobin HbSS or HbSC. A total of 79 children aged between 6 and 15 years with uncomplicated malaria attending the Ewim Health Centre were recruited between July 2015 and August 2017. A second set of 66 afebrile participants aged between 13 and 47 years, attending routine check-up at the Sickle Cell Clinic between June 2016 to April 2017 were also recruited into a cross sectional study. An additional set of 4 healthy volunteers aged between 25 and 35 years with HbAA RBCs were recruited from the NMIMR to supply blood for the in vitro parasite cultures and also serve as controls for the merozoite invasion assay.

The Sickle Cell Clinic at the Ghana Institute of Clinical Genetics, Korle-Bu is situated within the Accra metropolis of the Greater Accra Region of Ghana. The Ewim polyclinic is in the Cape Coast metropolis of the Central Region of Ghana.

### Sample acquisition and processing

Each study participant donated three drops of finger-pricked blood (~ 150 µL), which was used to prepare thick and thin blood smears, spot an HRP2-RDT and filter paper blood blots (Whatman^**®**^ #3, GE Healthcare, USA) for gDNA extraction and ABO blood grouping. Additionally, 2.5 mL of venous blood was collected from each participant into acid citrate dextrose (ACD) tubes for use in parasite cultures. All persons attending the various health facilities had their axillary temperature measured using a handheld digital thermometer.

Venous blood from the afebrile volunteers was separated by centrifugation and the plasma stored at − 20 °C. The blood cell pellets were washed twice with 2 volumes of incomplete parasite medium (iCPM: RPMI 1640 supplemented with 25 mM Hepes, 2 mM l-glutamine, 25 mM NaHCO_3_, 20 mM glucose, 5 μg/mL gentamycin, 50 μg/mL of hypoxanthine). The pelleted RBCs were finally resuspended in an equal volume of complete parasite media (CPM: iCPM supplemented with 0.5% Albumax II and 2% of normal human serum) and kept at 4 °C for no longer than 3 days. The whole blood collected from the children with uncomplicated malaria was processed as described for the healthy volunteers above. However, these RBCs were re-suspended at 3% haematocrit and used immediately to plate the ex vivo assay.

### ABO blood grouping

ABO blood group phenotyping was performed based on the forward tile method adopted from Karl Landsteiner’s haemagglutination protocol, using a commercially available kit (Accucare, Lab Care Diagnostics, India) according to manufacturer’s instructions. Briefly, three separate small drops (20 µL) of plasma from each individual were put on a glass slide. An equal volume of (20 µL) of anti-serum A, B or O (from the test kit) was individually added to the blood spot and mixed immediately. Blood group was scored after visually observing the mixture for evidence of agglutination within 2 min.

### Beta globin genotyping

The haemoglobin variant genotyping was performed using the PCR–RFLP protocol previously described by Danquah et al. [39] to detect the homo- and heterozygous A, C and S haemoglobin alleles. Amplification of a 358 bp region of the β globin gene was done in a 30 μL reaction volume using 200 nM of SC1F (5′-AGGAGCAGGGAGGGCAGGA-3′) and SC2R (5′-TCCAAGGGTAGACCACCAGC-3′) oligonucleotide primers. The reaction contained 1× GC rich buffer, 200 nM dNTP, 150 nM MgCl_2_, and 1 U of One Taq DNA polymerase (Thermo Fisher Scientific, UK). The cycling conditions were; initial denaturation at 94 °C for 5 min, followed by 35 cycles with 94 °C for 50 s, 64 °C for 50 s and 72 °C for 40 s; with a final extension at 72 °C for 5 min. The PCR products were subjected to digestion with *Mnl*I and *DdeI* restriction enzymes (New England BioLabs, UK) by incubating at 37 °C for 30 min to detected HbSC and HbSS genotypes. All PCR–RFLP products were resolved on 3% agarose gels and fragment sizes were used to determine the various Hb genotypes as previously described [39].

### In vitro parasite culture

A continuous culture of the *P. falciparum* NF54 strain was maintained at 2% haematocrit in complete parasite media (CPM: RPMI 1640 supplemented with HEPES, l-glutamine, NaHCO_3_, glucose, gentamycin and Albumax II). Parasites were cultured in vitro using a modified version of the method by Trager and Jensen [40]. Briefly, parasites were maintained in O+ red blood cells (RBCs), equilibrated in a blood gas environment of 94% nitrogen, 5% CO_2_, 1% oxygen (Air Liquide, Birmingham, UK) and maintained in an incubator at 37 °C with daily media change and periodic supplementation of uninfected RBCs to maintain the parasitaemia below 5% until the cultures had expanded enough to be used for an assay. At 5% parasitaemia, the culture was synchronized with 5% sorbitol [41] and then allowed to expand with daily media change and thin smear preparation to monitor parasite growth and development. The culture was harvested at high (~ 15%) schizont population and used for the invasion assays.

### Ex vivo gametocyte and asexual parasite culture of samples from children with uncomplicated malaria

An aliquot (50 µL) of packed washed RBCs from the children with uncomplicated malaria (samples from the cohort with RBCs containing HbAA, HbAS and HbAC) were maintained in culture at 37 °C in duplicate wells of a 12-well plate using CPM. For the gametocyte culture, the CPM in each well was supplemented with 50 mM of *N*-acetyl glucosamine (NAG) to prevent the expansion of the asexual parasite population and subsequent production of culture-induced gametocytes [37].

An asexual parasite culture was set up identical to that described for the gametocyte cultures. However, the media was not supplemented with NAG. This set up was used to serve as a control for the gametocyte assay and also to determine the ex vivo asexual parasite multiplication rate. The plates were placed in a Modular^®^ incubating chamber, gassed for 6 min with mixed gas (94% nitrogen, 5% CO_2_, 1% oxygen) and placed in an incubator set at 37 °C. Parasite media was changed and thin smear prepared daily for 6 days, however, unlike the in vitro culture, no RBCs were added during the course of culturing.

### Evaluation of blood smears

The thick and thin smears were processed according to standard protocol [42]. Briefly, the slides were air-dried, after which the thin smears were fixed in methanol. Thick and fixed thin smears were then stained with 10% Giemsa for 15 min, after which they were air-dried and subsequently viewed under 100× oil immersion microscope [43]. Two independent microscopists read each slide.

### Evaluation of merozoite invasion into RBCs from afebrile individuals using flow cytometry

Invasion assays were performed as previously described [27]. Briefly, to distinguish acceptor (target) cell (HbSS and HbSC) from residual RBCs in parasite inoculum, acceptor cells were stained with a cytoplasmic fluorescent stain; 5-(and-6)-carboxyfluorescein diacetate succinimidyl ester (5(6) CFDA-SE; 20 µM; Invitrogen), prior to assay plating. Schizont stage parasites were mixed with the acceptor cells in a 1:1 ratio at 2% haematocrit in 96-well titre plates. All experiments were setup in duplicates. Assays were incubated overnight at 37 °C in a blood gas environment of 94% nitrogen, 5% CO_2_, 1% oxygen (Air Liquide, Birmingham, UK). Assays were stained with 5 µM Hoechst (Hoechst 33342 Sigma Aldrich) after incubation to differentiate parasitized RBCs from uninfected ones. Invasion levels were determined by flow cytometry (LSR Fortessa X-20; BD) as described in other studies [25, 27]. Invasion levels in individual assays were determined by recording the proportion (in percentage) of RBCs that were dual positive for CFDA and Hoechst 33342. Invasion rates into RBCs containing HbSS and HbSC were then calculated by comparing their invasion levels to that of the control HbAA group, which was set at 100%.

### Data analysis

Parasite density was determined as the number of malaria parasites observed per 200 white blood cells on the thick smear, multiplied by 40 [44]. Parasitaemia measurements were determined as the percent of parasite-infected RBCs observed per 1000 RBCs on the thin smear. Parasite multiplication rate was defined as the fold-increase in asexual parasitaemia observed in cultures between D4 and D6. The production of early-stage (II) gametocytes on Day 4 was expressed as the percent of stage-specific infected RBCs counted per 20,000 RBCs [37].

BD FACSDIVA V8.0.1 was used to analyse the flow cytometry data. Invasion rate in the RBC variants was calculated as the percent of infected RBC contained in the patient blood (A) relative to that in the control blood (C) [(A/C) * 100)].

GraphPad Prism 5.01 was used for all the statistical analysis, including descriptive statistics, *T* test, Kruskal–Wallis test and Dunn’s Multiple Comparison test. Dunn’s Multiple Comparison test was performed only when the Kruskal–Wallis test identified a significant difference. Parasite density in samples containing HbAA, HbAC and HbAS on D0 was compared using Kruskal–Wallis test followed by Dunn’s Multiple Comparison test. Parasite multiplication rates and gametocyte densities were compared using Kruskal–Wallis test. *p* ≤ 0.05 was considered significant.

## Results

### Demographics of study participants

The study recruited 79 children aged between 6 and 15 years old with uncomplicated malaria, out of which 24% (19/79) had HbAC, 19% (15/79) had HbAS and 60% (45/79) had HbAA (Table 1). The HRP2-RDT positivity rate in the children with uncomplicated malaria was 100%. The median parasite density in the HbAC and HbAS children were similar but the median parasite density for children with HbAC was significantly lower than those with the HbAA genotype (Table 1).

**Table 1:** Demographic characteristics of participants with uncomplicated malaria

UM	HB genotype		
	HbAA (n = 45)	HbAC (n = 19)	HbAS (n = 15)
Sex (F/M)	20/22	11/7	5/10
Age (years)			
Min–max	1–15	1–15	1–12
Median (IQR)	6 (3–8)	5 (4–9.5)	7 (5–8.25)
RDT (%)	100	100	100

Uncomplicated malaria—axillary temperature at or above 37.5 °C and presence of malaria parasites by microscopy; afebrile—axillary temperature below 37.5 °C and absence of malaria parasites by microscopy as well as negative by a malaria RDT

*F* female, *M* male, *BG* blood group, *PD* Parasite density measured as parasite, *p* per microlitre; *min* minimum, *max* maximum, *IQR* interquartile range (25–75%); *RDT* HRP2 based malaria rapid diagnostic test positivity rate, *Temp* axillary temperature, *ND* not done, *NP* no parasite observed by microscopy

The second cohort comprised of 66 afebrile participants aged between 13 and 55 years. There were 53% (35/66) of the afebrile study participants that had HbSS and another 47% (31/66) had HbSC (Table 2). The number of males and females in both cohorts was similar (Tables 1 and 2). The HRP2-RDT positivity rate and parasite densities of the afebrile participants were both 0.

**Table 2:** Demographic characteristics of the afebrile study participants

Afebrile	HB genotype	
	HbSS (n = 35)	HbSC (n = 31)
Sex (F/M)	16/19	17/14
BG (O/A/B)	24/7/4	14/6/10
Age (years)		
Min–max	13–55	14–47
Median (IQR)	24 (20–30)	26 (19–55)

Uncomplicated malaria—axillary temperature at or above 37.5 °C and presence of malaria parasites by microscopy; afebrile—axillary temperature below 37.5 °C and absence of malaria parasites by microscopy as well as negative by a malaria RDT

*F* female, *M* male, *BG* blood group, *PD* Parasite density measured as parasite, *p* per microlitre; *min* minimum, *max* maximum, *IQR* interquartile range (25–75%); *RDT* HRP2 based malaria rapid diagnostic test positivity rate, *Temp* axillary temperature, *ND* not done, *NP* no parasite observed by microscopy

### Parasite density in children with uncomplicated malaria

**Fig. 1:**
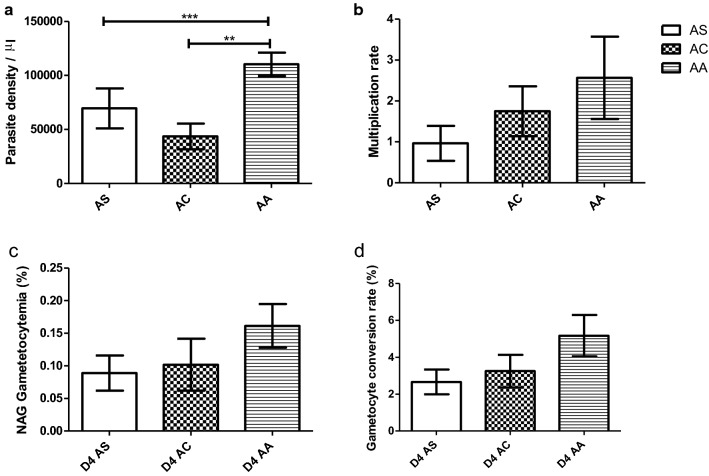
Ex vivo parasite development in normal and variant Hb erythrocytes. **a** Parasite density on D0, **b** asexual parasite multiplication rates, **c** gametocytaemia on D4 and **d** gametocyte conversion rate. **a** Giemsa-stained thin blood smears prepared from AS n = 15, AC n = 19 and AA n = 45 D0 samples were used to determined asexual parasitaemia. **b** The fold increase in asexual parasite densities in samples counted on D6 were compared to that counted in the same culture on D4 from AS n = 12, AC n = 14 and AA n = 40 samples. **c** Gametocyte densities in samples from AS n = 12, AC n = 14 and AA n = 43 examined on D4. **d** D4 Gametocyte conversion rate, represented by the % of the D0 ring stage parasites that develop into early stage (II) gametocytes by D4 from AS n = 12, AC n = 14 and AA n = 43 samples. Parasite infected RBCs in the thin blood smears were counted against 1000 RBCs for asexual parasite density estimation and 20,000 RBCs for gametocyte densities

The asexual parasite densities in the samples collected from the uncomplicated malaria patients ranged from 2604 parasites/μL to 249,960 parasites/μL, with a median (IQR) of 81,148 ± 6648 parasites/μL. The samples from participants with HbAA had the highest parasite densities, with a median (IQR) of 88,716 (43,740–164,775) parasites/μL and the least was identified in samples with HbAC, which had a median (IQR) parasite density of 24,615 (17,043–32,804) parasites/μL (Table 3). The parasite density in samples with HbAS was similar to that of HbAC, which were both significantly lower than the parasite density in HbAA (Dunn’s multiple comparison test, p < 0.001 for HbAA and HbAC and p < 0.01 for HbAA and HbAS (Fig. 1a).

**Table 3:** Malariaometric indices of children with uncomplicated malaria

	HbAA	HbAC	HbAS
D0 PD (p/μL)			
Count (n/N)	100% (45/45)	100% (19/19)	100% (15/15)
Median (IQR)	88,716 (43,740–164,775)	24,615 (17,043–32,804)	23,540 (9130–122,760)
Min–max	3910–249,960	2604–226,830	3621–248,116
D0 AS			
Count (n/N)	100% (45/45)	100% (19/19)	100% (15/15)
Median (IQR)	3.2 (1.93–4.28)	3.7 (0.78–6.1)	2.2 (1.4–5.7)
Min–max	0.90–21	0.78–13.30	0.29–7.1
D4 ES			
Count (n/N)	95.5% (43/45)	73.7% (14/19)	80% (12/15)
Median (IQR)	0.08 (0.02–0.25)	0.05 (0.01–0.12)	0.03 (0.01–0.10)
Min–max	0.004–1.39	0.009–0.34	0.004–0.486

Count stated as a percent followed by a fraction

*PD* parasite density measured as parasites, *p* per microlitre, *AS* asexual *P. falciparum* parasites, *ES* early stage *P. falciparum* gametocytes, *min* minimum, *max* maximum, *IQR* interquartile range (25–75%), *n* number positive, *N* total number present

### Parasite multiplication rate in RBCs from children with uncomplicated malaria

The fold-increase of asexual parasite infected RBCs (multiplication rate) within each culture was measured in the control cultures (grown in the absence of NAG) of HbAA, HbAS and HbAC between D4 and D6. The asexual parasite multiplication rates ranged from a median (IQR) of 2.3 (1.2–3.2) in HbAA, 1.55 (1.2–2.55) in HbAC and 1.35 (0.85–2.0) in HbAS cells (Fig. 1b, Additional file 1: Table S1). However, no statistical significant difference (Kruskal–Wallis test, p = 0.056) was observed among the multiplication rates between erythrocytes containing normal HbAA and the variant Hb (HbAS and HbAC) genotypes (Fig. 1b, Additional file 1: Table S1).

### Ex vivo gametocyte development in RBCs from children with uncomplicated malaria

Gametocyte densities in the cultures from the symptomatic participants were determined after four (D4) days of continuous culture in the presence of NAG. The NAG in the media ensures that only gametocytes (stage II) produced from ring-stage parasites that were sexually committed at the time of the initial culture set up were determined. Gametocyte prevalence on D4 in all the three different RBC Hb types (HbAA, HbAS and HbAC) were similar (Kruskal–Wallis test, p = 0.166), although cultures containing HbAA produced the highest gametocytaemia (median, IQR) of, 0.08, 0.02–0.25, while the lowest gametocytaemia of 0.03, 0.01–0.10) were found in HbAS cultures (Fig. 1c, Additional file 1: Table S1). The ratio between D4 gametocytemia and D0 parasitaemia, which represents the gametocyte conversion rate in HbAA, HbAS and HbAC erythrocytes was also similar (Fig. 1d, Additional file 1: Table S1).

### Merozoite invasion rates in RBCs from afebrile individuals

**Fig. 2:**
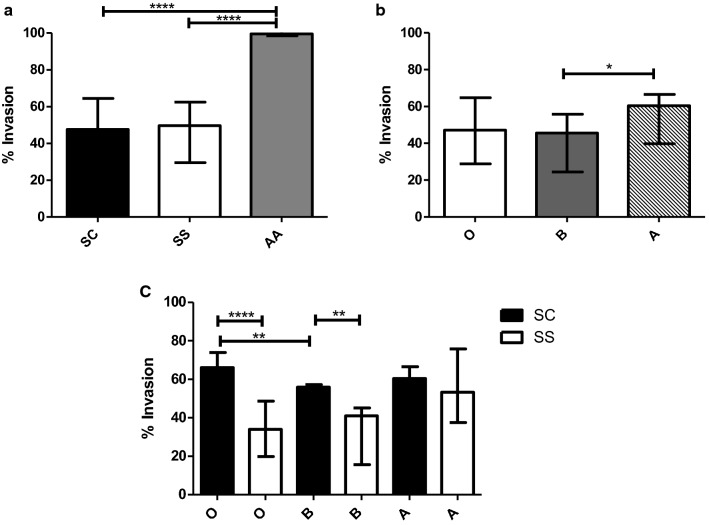
Parasite invasion into normal and variant Hb erythrocytes. **a** Invasion of NF54 into erythrocytes with variant (HbSS n = 35 and HbSC n = 31) and normal (HbAA n = 4) Hb genotypes. The assay was performed in duplicate. **b** Invasion rates in HbSS and HbSC samples with different blood groups, ‘O’ n = 38, ‘B’ n = 14 and ‘A’ n = 14. **c** Invasion in HbSS (‘O’ n = 24, ‘A’ n = 7, ‘B’ n = 4) and HbSC (‘O’ n = 14, ‘A’ n = 7, ‘B’ n = 10) RBCs categorized according to blood group antigen. The data in the graphs represent the median and the error bars are the interquartile range

The median (IQR) invasion rates into RBCs containing HbSS and HbSC was 49.6% (29.6–62.4) and 47.6% (30.7–64.5) respectively, which were both significantly lower (Kruskal–Wallis test, p < 0.0001) than invasion into RBCs containing the normal HbAA genotype (median (IQR) of 99.5% (98.6–101). Dunn’s multiple comparison for HbSS vs HbAA and HbSC vs HbAA, p = 0.001 for both combinations) (Fig. 2a, Additional file 1). However, no significant difference was observed between the invasion rates into RBCs containing HbSC relative to HbSS (Mann–Whitney test, *p *= 0.977).

The influence of varying blood group antigen on merozoite invasion rates into erythrocytes containing variant Hb genotypes, HbSC and HbSS combined was determined by sorting the invasion rate data for the variant RBCs according to blood group antigen. The median (IQR) invasion rates into the various blood groups were 60.35% (39.8–66.5) in blood group A, 45.6% (24.4–55.8) in blood group B and 47.1% (28.8–64.8) in blood group O. Reduced merozoite invasion rates was observed in RBCs with blood groups B and O relative to blood group A, but the difference was only statistically significant between RBCs containing blood group B and A (Mann–Whitney test, *p *= 0.007) (Fig. 2b, Additional file 1: Table S1).

To understand the individual contribution of blood group antigen to merozoite invasion into each of the groups of Hb variants (HbSC and HbSS separately), merozoite invasion for each blood group antigen was analysed separately for RBCs containing the HbSC and HbSS. Merozoite invasion rates in HbSS containing red blood cells were similar in all of the ABO blood groups (Kruskal–Wallis test, p = 0.054). Among the HbSC containing RBCs, invasion rates were significantly different (Kruskal–Wallis test, p = 0.002), with invasion rates reduced in blood group B containing RBCs relative to the blood group O (Dunn’s Multiple comparison test, *p *< 0.01; Mann–Whitney test, U = 58.5, p = 0.0003) (Fig. 2c, Additional file 1: Table S1).

The median merozoite invasion rates in blood groups O and B were significantly higher in the HbSC group (66.06% and 55.87% respectively) compared to blood groups O and B in the HbSS group (33.975 and 40.98% respectively) (Mann–Whitney test, U = 174.5, p < 0.0001 for blood group O and U = 36.5, p = 0.919 for blood group B). Merozoite invasion rates in blood group A RBCs with HbSC (60.35%) was similar (Mann–Whitney test, U = 77.5, p < 0.0001 to those in HbSS group (53.32%) (Fig. 2c, Additional file 1: Table S1).

## Discussion

A number of studies have reported the sickle cell trait and haemoglobin C to provide anti-disease immunity against both severe and uncomplicated malaria [3, 4, 10, 45–48]. A variety of mechanisms governing this phenomenon have been suggested. This study sought to characterize RBC invasion into RBCs containing a haemoglobinopathy (a variant Hb genotype) as well as compare gametocyte development and asexual parasite multiplication rates in RBCs containing HbAA and other haemoglobinopathies.

During a natural infection of uncomplicated malaria, children with HbAC and HbAS had significantly lower parasite densities than children with both HbAA, supporting previous reports which suggest a protective effect of haemoglobin variants HbAC and HbAS against high parasitaemia and severe disease [7, 49]. High parasite densities found in children with HbAA RBCs relative to RBCs with haemoglobinopathies has been previously reported [9, 45, 47].

In contrast to asexual parasitaemia, RBCs with HbC and HbS genotypes have been reported to contain high numbers of stage V gametocytes [50]. However, none of the children in this study had microscopically detectable stage V gametocytes on D0, even though > 70% of the subjects had circulating ring stage parasites that differentiated into stage II–III by D4 of ex vivo culture. Few studies have also identified a large proportion of asymptomatic individuals [51, 52] as well as symptomatic malaria patients [53], irrespective of erythrocyte Hb genotype or blood group, to harbor submicroscopic densities of gametocytes. Although the initial D0 parasitaemias were lower in HbAC and HbAS subjects, no significant difference was observed between gametocyte densities in HbAC and HbAS RBCs relative to HbAA RBCs. This could be due to the presence of a higher initial number of gametocyte committed ring stage parasites in the HbAC and HbAS relative to the parasite population in the HbAA RBCs. However, this claim would need to be validated by real time PCR as gametocyte committed rings and asexual ring stage parasites are indistinguishable by microscopy [37].

The present study affirm observations that RBCs with HbC genotype have low invasion capabilities compared to normal HbAA genotype [54]. The data further suggests that the erythrocytic mechanism of the anti-disease protection is associated with invasion in the abnormal haemoglobin variants and not only due to the intra-erythrocytic metabolic processes as previously suggested [3]. The ‘O’ blood group has been associated with protection against severe forms of malaria relative to non-‘O’ blood groups because the glycoproteins of non-‘O’ blood groups serve as receptors for a number of biological processes associated with malaria, including merozoite invasion and rosetting in the parasite [6, 48]. In this study, blood group B was associated with lower merozoite invasion rates compared to blood group O in the heterozygous HbSC RBCs but not in the homozygous HbSS cells in vitro. This observation suggests that the anti-malaria protection conferred by RBC variants is possibly dependent on the interaction of multiple conditions.

## Conclusions

Parasite invasion rate into erythrocytes is dependent on both erythrocyte blood group antigen and haemoglobin genotype, as blood group O and B provided protection via reduced merozoite invasion in RBCs containing HbSS relative to HbSC. Regardless of haemoglobin type, greater than 70% malaria patients had circulating ring stage parasites that differentiated into stage II gametocytes in 4 days.

## Supplementary information

**Additional file 1: Table S1.** Results of in vitro merozoite invasion rates, asexual parasite multiplication rate and gametocyte conversion of ex vivo cultures.

## Data Availability

All data generated or analysed during this study are included in this published article [and its additional information files].

## Abbreviations

RBC: Red blood cell
Hb: Haemoglobin gene (beta globulin gene)
HbS: Haemoglobin S
HbC: Haemoglobin C
G6PDd: Glucose 6 phosphate dehydrogenase deficiency
CPM: Complete parasite media
iCPM: Incomplete parasite media
NAG: *N*-acetyl glucosamine
PD: Parasite density
