# Supplementary material for: Comparative analysis of asexual and sexual stage Plasmodium falciparum development in different red blood cell types
Source: Malar J. 2020 Jun 5;19:200. doi: 10.1186/s12936-020-03275-9 (PMC7275330; doi:10.1186/s12936-020-03275-9)
Supplement: Supplementary file 1 — Additional file 1: Table S1. Results of in vitro merozoite invasion rates, asexual parasite multiplication rate and gametocyte conversion of ex vivo cultures. [file 12936_2020_3275_MOESM1_ESM.docx]

Table S1: Merozoite invasion rates asexual multiplication rate and gametocyte conversion of ex-vivo cultures.

|  |  |  | Min | Max | Median | IQR | p value |
| --- | --- | --- | --- | --- | --- | --- | --- |
|  | **HBB** | **SS** | 49.7 | 91.7 | 49.7 | 29.6 - 62.4 | p<0.0001 |
|  |  | **SC** | 47.6 | 64.6 | 47.6 | 30.7 - 64.5 |  |
| **1** |  | **AA** | 99.5 | 101.6 | 99.5 | 98.6 - 101 |  |
|  |  |  |  |  |  |  |  |
|  | **BG**  **(SS + SC)** | **O** | 9.5 | 85.6 | 47.1 | 28.8 - 64.8 | p=0.036 |
| **2** |  | **B** | 10.6 | 61.8 | 45.6 | 24.4 - 55.8 |  |
|  |  | **A** | 11.6 | 92.4 | 60.35 | 39.8 - 66.5 |  |
|  |  |  |  |  |  |  |  |
|  | **BG O** | **SC** | 14.8 | 85.6 | 66.1 | 60.5 - 73.9 | U=174.5, p<0.0001 |
|  |  | **SS** | 9.5 | 79.4 | 33.97 | 19.8 - 48.6 |  |
|  | **BG B** | **SC** | 23.3 | 61.8 | 55.8 | 35.6 - 57.2 | U=36.5, p=0.005 |
| **3** |  | **SS** | 10.6 | 55 | 41 | 15.6 - 45.1 |  |
|  | **BG A** | **SC** | 11.6 | 92.4 | 60.4 | 42.1 - 66.5 | U=77.5, p=0.919 |
|  |  | **SS** | 34.9 | 81.4 | 53.3 | 37.6 - 75.8 |  |
|  |  |  |  |  |  |  |  |
|  | **SC** | **O** | 14.8 | 85.6 | 66.1 | 60.5 - 73.9 | p=0.002 |
| **4** |  | **B** | 23.3 | 61.8 | 55.8 | 35.6 - 57.2 |  |
|  |  | **A** | 11.6 | 92.4 | 60.4 | 42.1 - 66.5 |  |
|  |  |  |  |  |  |  |  |
|  | **SS** | **O** | 9.5 | 79.4 | 33.97 | 19.8 - 48.6 | p=0.054 |
| **5** |  | **B** | 10.6 | 55 | 41 | 15.6 - 45.1 |  |
|  |  | **A** | 34.9 | 81.4 | 53.3 | 37.6 - 75.8 |  |
|  |  |  |  |  |  |  |  |
|  | **MR** | **AA** | 0.313 | 4.5 | 2.3 | 1.2 - 3.2 | p=0.056 |
| **6** |  | **AC** | 0.5 | 3.43 | 1.55 | 1.2 - 2.55 |  |
|  |  | **AS** | 0.46 | 2.47 | 1.35 | 0.85 - 2.0 |  |
|  |  |  |  |  |  |  |  |
|  | **GR** | **AA** | 1 | 8 | 3 | 1.5 - 4 | p=0.476 |
| **7** |  | **AC** | 1 | 11 | 4.5 | 1 - 6.5 |  |
|  |  | **AS** | 1 | 11 | 5.5 | 1 - 10.25 |  |

Invasion rates are presented as % Invasion. Statistical analysis of samples obtained from the afebrile study participants (**1-5**): P values are based on Kruskal-Wallis tests for comparison of invasion into the 3 HB genotypes (**1**:AA, SC and SS), the three blood groups (A, B and A) for erythrocytes with HbSS and HbSC combined (**2**) or individually (**4** and **5**). The p values for the comparison of invasion into the same blood group of HbSS and HbSC (**3**) erythrocytes was determined using Mann-Whitney tests.

Statistical analysis of samples obtained from the children with uncomplicated malaria (6&7): P values are based on Kruskal-Wallis tests for comparison of merozoite invasion rates (**MR**) and gametocyte conversion rates (**GR**) into erythrocytes containing the 3 HB genotypes.
